# Stress Intensity Factor (SIF) Solutions and Fatigue Crack Paths in Eccentric Circumferentially Cracked Round Bar (CCRB) in Tension

**DOI:** 10.3390/ma16041728

**Published:** 2023-02-20

**Authors:** Jesús Toribio, Juan-Carlos Matos, Beatriz González

**Affiliations:** Fracture & Structural Integrity Research Group (FSIRG), University of Salamanca (USAL), E.P.S., Campus Viriato, Avda. Requejo 33, 49022 Zamora, Spain

**Keywords:** circumferentially cracked round bar (CCRB), eccentricity of circular ligament, fatigue crack paths, *no contact*, *partial contact*, *full contact*

## Abstract

In this paper, a numerical modeling was developed to study (on the basis of the Paris law) the fatigue propagation paths of eccentric external (outer) cracks in circumferentially cracked round bars (CCRB) subjected to a cyclic type of loading in the form of either *remote tensile loading* or *imposed axial displacement*. Results show how the eccentricity (in relation to the wire axis) of the circular resistant ligament increases with the growth of outer circumferential cracks by subcritical fatigue mechanisms. This phenomenon is more pronounced when the solicitation consists of a remote tensile loading than when it is an axial displacement, when the initial eccentricity of the ligament increases (for a given initial diameter), and when the Paris exponent characteristic of the material rises. The paper also analyzes in depth the different situations regarding contact between crack faces during subcritical cyclic fatigue propagation, covering a wide range of cases including *no contact*, *partial contact*, and *full contact* depending on the ligament diameter (during the process of fatigue crack advance) and the relative eccentricity of the annular crack that loses its axial symmetry in relation to the round bar (cylinder) axis. In addition to the fatigue crack path study, *closed-form* stress intensity factor (SIF) solutions for the considered geometry (a cylinder with an outer annular crack) are provided in the form of third-degree polynomial expressions as a function of the ligament diameter and the crack eccentricity (both in dimensionless terms).

## 1. Introduction

In the case of metallic materials, circumferentially cracked round bar (CCRB) specimens, i.e., cylindrical specimens containing external (outer) circumferential cracks, are an interesting scientific alternative to the well-known standard specimens considered in the ASTM E399, *Standard Test Method for Linear-Elastic Plane-Strain Fracture Toughness K_Ic_ of Metallic Materials*, cf. [1] for measuring the fracture toughness *K*_IC_ of materials [2,3,4]. They have been successfully used to study cyclic fatigue crack propagation [5,6], stress corrosion cracking [7], and corrosion-fatigue [8].

From the experimental point of view, the main advantages of the aforesaid CCRB specimens are the low times for preparing the test specimens and the short duration of the experiments themselves [6], with the consequent saving of time. In addition, they exhibit fundamental scientific advantages in the purely conceptual framework, such as the achievement of a triaxial stress distribution in the vicinity of the crack tip, which makes a plane strain condition take place even for small specimen diameters [2], thereby providing a more reliable value of the fracture toughness of the material, more than a simply critical value of the stress intensity factor (SIF).

Nevertheless, the described CCRB specimens exhibit some disadvantages, such as their tendency to produce the appearance of certain specimen eccentricity when the crack grows [2], with the subsequent loss of axial symmetry of the specimen and potential problems of adequate experimental alignment of the sample with the grips of the testing machine. Such an undesirable phenomenon has been related to either the described non-symmetric geometry of the sample, to the testing grips themselves, or even to the presence of a residual stress field in the sample or the non-uniform properties of the material [4].

In the scientific literature, there are SIF solutions in CCRBs subjected to axial tensile loading, containing either the more conventional symmetric situation [9,10,11] or the more realistic non-symmetric configuration with eccentric annular outer cracks [12,13]. In the latter case, additional bending stresses appear as a consequence of the lack of symmetry, and a new issue arises in the matter of possible contact between faces, with a wide range of situations covering *no contact*, *partial contact*, and *full contact*. 

The sample eccentricity rises when the crack growths by fatigue (cyclic) loading [14,15,16] with the associated increasing loss of axial symmetry. In addition, the increase in initial eccentricity or the initial misalignment in the specimen produces a higher increase in eccentricity during fatigue propagation [15,16], thereby diminishing the time required for reaching the critical situation.

This paper provides *closed-form* SIF solutions for CCRBs in the form of third-degree polynomial expressions as a function of the ligament diameter and the crack eccentricity (both in dimensionless terms), covering a wide range of geometries and the two loading schemes considered in the present paper, namely *remote tensile loading* and *imposed axial displacement*.

In addition, the present paper analyzes the fatigue crack path evolution in a CCRB subjected to either remote cyclic tensile loading or imposed cyclic axial displacement, going further in the brief analysis initiated recently in a paper included in conference proceedings [17]. 

## 2. Numerical Modeling

**Figure 1:**
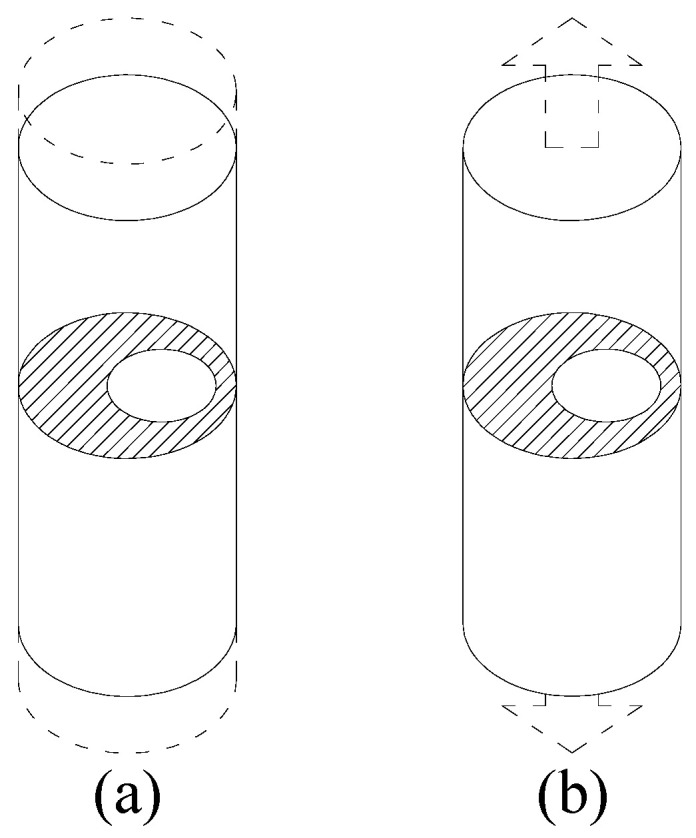
Round bar with an external circular crack for: (**a**) imposed axial displacement; (**b**) remote (axial) tensile loading.

Numerical modeling was performed to study the crack tip stress and strain fields and the fatigue crack propagation paths associated with external annular cracks in round bars subjected to imposed axial displacement (Figure 1a) or remote (axial) tensile loading (Figure 1b). The specimen length is equal to twenty times its diameter *D*.

**Figure 2:**
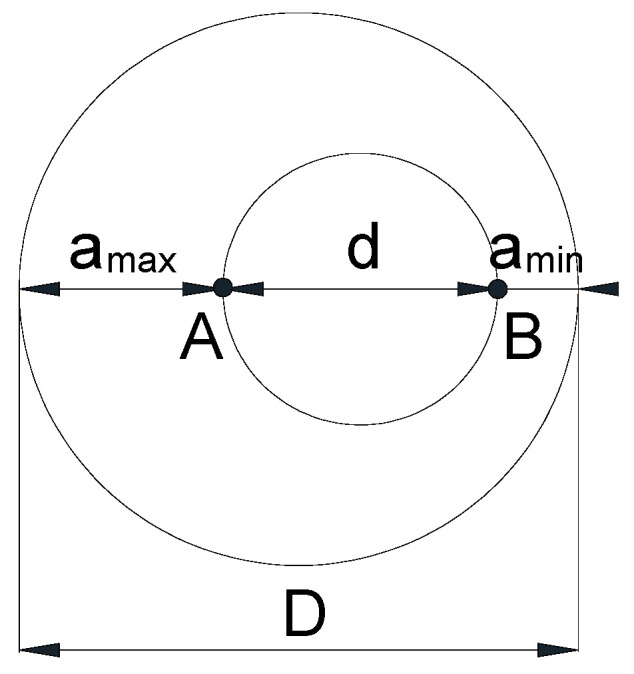
Details of the cracked area.

The eccentric CCRB geometry with an annular external crack (Figure 2) was characterized by the following parameters: round bar diameter *D*, maximum crack depth *a*_max_ (related to point A), minimum crack depth *a*_min_ (linked to point B), ligament diameter *d*, and ligament eccentricity *ε* (or, in other words, crack eccentricity, since the loss of axial symmetry applies to both the internal circular ligament and to the external annular circumferential crack), i.e.,

(1) $$\varepsilon =\frac{a_{\max}-a_{\min}}{2}$$

The finite element method (FEM) analysis, performed with MSC. Marc 2012 software, has been used to simulate the eccentric CCRB subjected to tension (for imposed axial displacement and for remote tensile loading), as explained in detail in a previous paper [13]. Only a quarter of the bar was modeled (taking advantage of the problem symmetry), and 20-node isoparametric hexahedral elements were used, with the nodes closest to the crack front shifted to the quarter-point position to model the crack tip singularity. A convergence study of the mesh size was performed, decreasing the size of the elements in the area close to the crack front, and the SIF *K*_I_ was obtained from the *J*-integral calculation under plane strain conditions.

The basic hypothesis of the modeling is that the crack advances by fatigue cyclic loading while the crack front maintains a circular shape, following the Paris–Erdogan Law [18]:

(2) $$\frac{da}{dN}=C\Delta K^{m}$$

where *C* and *m* are the Paris parameters (characteristics of the material). In the calculation of the fatigue crack path, carried out in successive iterations, only points A and B at the circular crack front were taken into account.

The advance Δ*a*_A_ of the point A is the same throughout the calculation, while the progress Δ*a*_B_ of the point B is performed with the Paris law as:

(3) $$\Delta a_{B}=\Delta a_{A}{\left[\frac{K_{\mathrm{IB}}^{*}}{K_{\mathrm{IA}}^{*}}\right]}^{m}$$

where *K*_I_^*^ is the dimensionless SIF:

(4) $$K_{I}^{*}=\frac{K_{I}}{\sigma \sqrt{\pi D}}$$

## 3. Numerical Results

### 3.1. Stress Intensity Factor (SIF)

**Figure 3:**
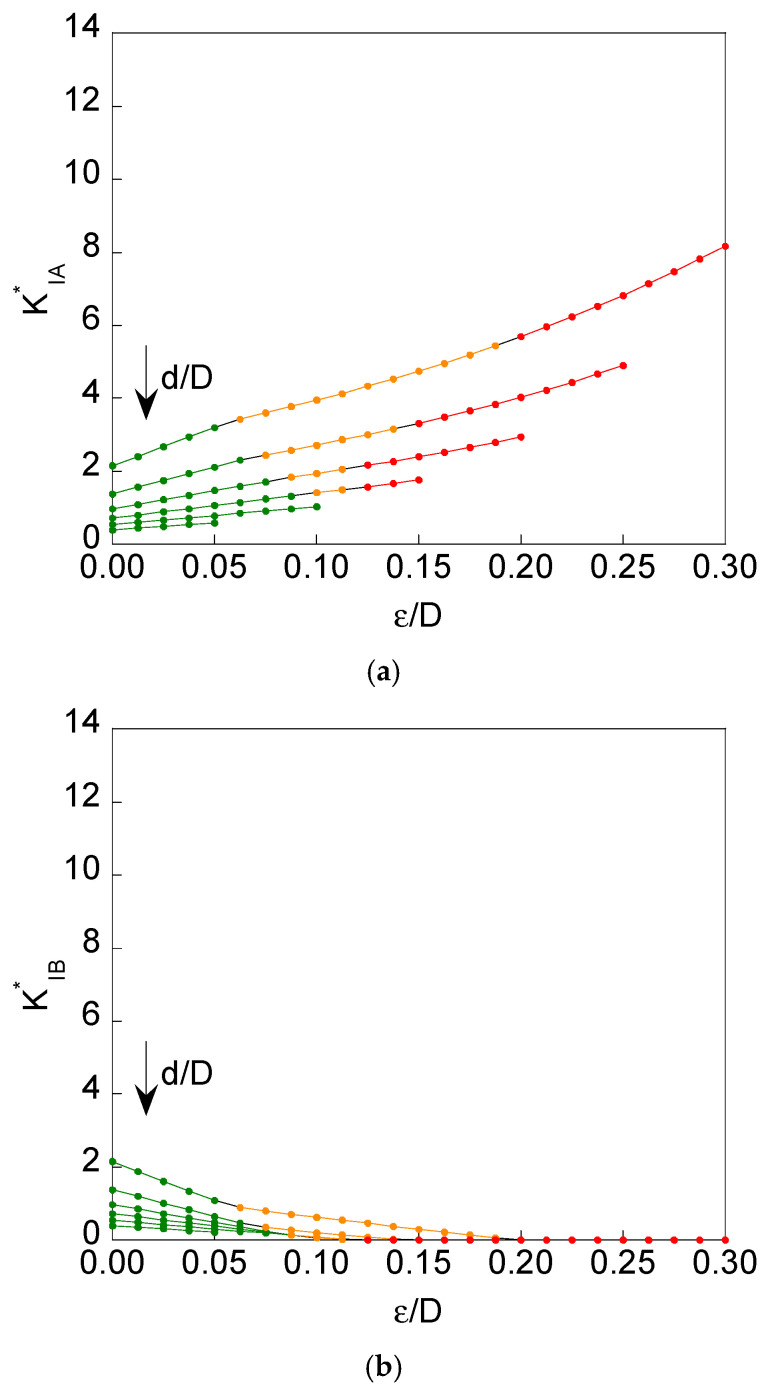
For *imposed axial displacement* and *d*/*D* = {0.3, 0.4, 0.5, 0.6, 0.7 and 0.8}: (**a**) maximum dimensionless SIF K^*^_IA_ vs. relative ligament eccentricity *ε*/*D;* (**b**) minimum dimensionless SIF K^*^_IB_ vs. relative ligament eccentricity *ε*/*D* (green: *no contact*; orange: *partial contact*; red: *full contact*).

**Figure 4:**
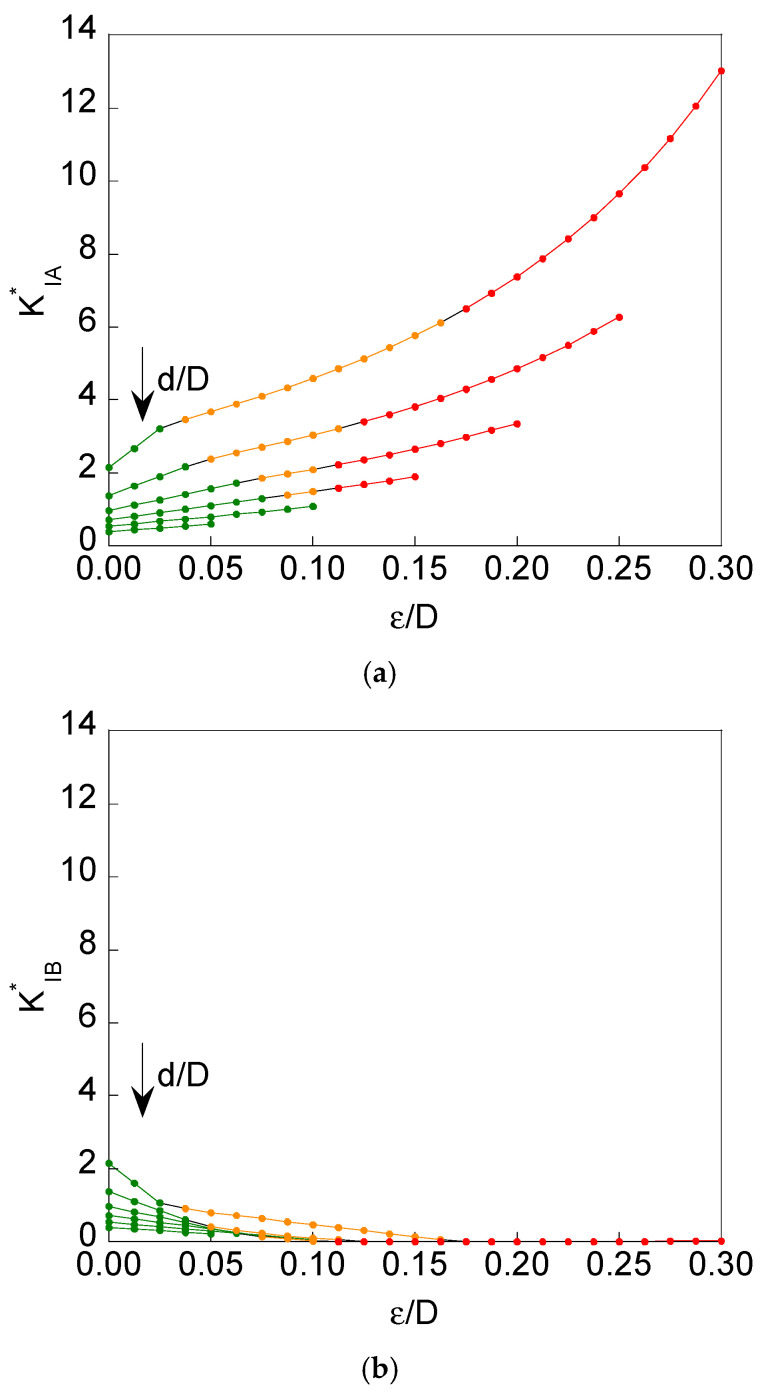
For *remote tensile loading* and *d*/*D* = {0.3, 0.4, 0.5, 0.6, 0.7 and 0.8}: (**a**) maximum dimensionless SIF K^*^_IA_ vs. relative ligament eccentricity *ε*/*D;* (**b**) minimum dimensionless SIF K^*^_IB_ vs. relative ligament eccentricity *ε*/*D* (green: *no contact*; orange: *partial contact*; red: *full contact*).

The dimensionless SIF *K*_I_^*^ associated with points A and B of the crack front was calculated as described above. Figure 3 and Figure 4 show the dimensionless SIF solutions at points A and B of the crack front (maximum dimensionless SIF K^*^_IA_ and minimum dimensionless SIF K^*^_IB_) for the specific values *d*/*D* = {0.3, 0.4, 0.5, 0.6, 0.7, 0.8} and for *ε*/*D* from 0 (*symmetrical case ε* = 0) to (0.5(1-*d*/*D*)-0.05) with increments of 0.0125.

It is seen that a magnification of SIF values is achieved at point A (K^*^_IA_) in the case of remote tensile loading (when compared to imposed axial displacement), mainly for the highest eccentricity value (*ε/D* = 0.30). The cause is the *bending effect* that appears when the axial loading is in remote tension, i.e., in the absence of displacement constraint, thereby allowing the rotation of the sample ends, which is favoured by the geometric eccentricity of the resistant ligament in relation to the bar axis. On the other hand, such a rotation is prevented in the case of imposed axial displacement, since the latter implies a constraint in the vicinity of the sample ends, a region in which rotation is impossible, thus reducing the bending effect and therefore diminishing the SIF value at point A (K^*^_IA_) when compared to the case of remote tensile loading, in which the aforesaid bending effect is quite pronounced. 

For *lower levels of eccentricity* (for all the values of the specimen ligament diameter), *no contact* between crack faces appears, and the situation remains closer to the symmetric case where there is no dissimilarity between the crack-tip stress-strain fields in the vicinity of point A and in the near-tip area of point B (see Figure 2). This happens for both loading scheme situations considered in the present paper, namely, imposed axial displacement and remote tensile loading.

**Figure 5:**
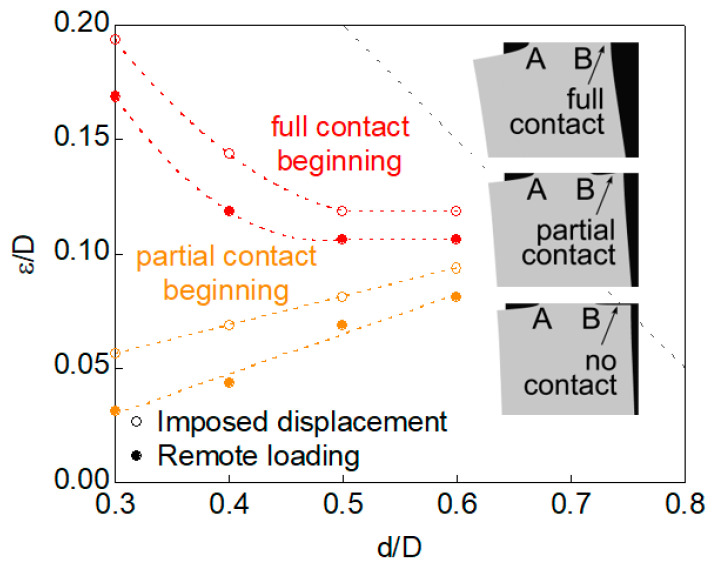
Curves *ε*/*D* vs. *d*/*D* for the beginning of *partial contact* and *full contact*.

For *higher levels of eccentricity* (for small values of the specimen ligament diameter), *contact* arises between crack faces in the vicinity of point B (see Figure 2) as a consequence of the bending effect appearing in the specimen, and the situations differ more from the symmetric case in such a manner that there is a certain dissimilarity between the crack-tip stress-strain fields in the vicinity of point A and in the near-tip area of point B (see Figure 2). The described phenomenon of contact appears for both loading scheme situations considered in the present paper, namely, imposed axial displacement and remote tensile loading, and it takes place in two sub-forms:

(i) *partial contact* in the cases of values of relative ligament diameter *d*/*D* ≤ 0.6 and from certain eccentricity level *ε*/*D* (this level being higher as *d*/*D* increases, following a more or less quasi-linear relationship, as shown in Figure 5 in the matter of partial contact beginning).

(ii) *full contact* in the cases of values of relative ligament diameter *d*/*D* ≤ 0.6 (the same interval as in the case of a partial contact) and from certain eccentricity level *ε*/*D* (this level being lower as *d*/*D* increases, following curvilinear relationship tending to a horizontal asymptotic level, as shown in Figure 5 in the matter of full contact beginning).

It is important to emphasize that in this case of full contact, the dissimilarity between the crack-tip stress-strain fields is maximum since the maximum SIF is achieved at point A (very intense near-tip field as a consequence of specimen bending), whereas at the opposite point B the near-tip field is null, and the SIF is zero.

With regard to the differences between both loading scheme situations considered in the present paper, namely, imposed axial displacement and remote tensile loading, and in the matter of the phenomenon of contact, it is possible to say that the event of contact is more likely (and more pronounced) in the case of remote tensile loading than in the situation of imposed axial displacement, since the bending effect has a more important role in the former than in the latter, i.e., an axial displacement, in a certain sense, prevents (or at least, diminishes) the bending effect, thereby requiring higher levels of eccentricity to generate both partial and full contact (when compared to the case of remote tensile loading, which enhances bending and promotes both partial and full contact even for lower levels of specimen eccentricity).

The numerical results with regard to the dimensionless SIF *K*_I_^*^ were fitted to polynomial expressions of the third degree using a least squares method as a function of the ligament diameter *d*/*D* and the crack eccentricity *ε*/*D* (both in dimensionless terms), covering a wide range of geometries, thereby providing a high level of generality to the paper since such analytical expressions are applicable to any fracture mechanics, damage tolerance, and structural integrity analysis of CCRB.

The closed-form dimensionless SIF solutions for the different cases (*imposed axial displacements* and *remote tensile loading*; *no contact* and *partial contact*) are given at both crack front points A and B as follows:

- For *imposed axial displacement* and *no contact*:

(5) $$\begin{array}{l} K_{{}_{\mathrm{IA},u\;\left[\mathrm{no}\;\mathrm{contact}\right]}}^{*}=-18.73{\left(d/D\right)}^{3}+63.13{\left(d/D\right)}^{2}\left(\varepsilon /D\right)+6.01\left(d/D\right){\left(\varepsilon /D\right)}^{2}+\\ +36.24{\left(\varepsilon /D\right)}^{3}+38.20{\left(d/D\right)}^{2}-103.69\left(d/D\right)\left(\varepsilon /D\right)-6.89{\left(\varepsilon /D\right)}^{2}-\\ -27.36\left(d/D\right)+46.36\left(\varepsilon /D\right)+7.42\\ K_{{}_{\mathrm{IB},u\;\left[\mathrm{no}\;\mathrm{contact}\right]}}^{*}=-15.33{\left(d/D\right)}^{3}-74.38{\left(d/D\right)}^{2}\left(\varepsilon /D\right)-48.08\left(d/D\right){\left(\varepsilon /D\right)}^{2}+\\ +112.58{\left(\varepsilon /D\right)}^{3}+32.60{\left(d/D\right)}^{2}+118.34\left(d/D\right)\left(\varepsilon /D\right)+15.93{\left(\varepsilon /D\right)}^{2}-\\ -24.46\left(d/D\right)-50.33\left(\varepsilon /D\right)+6.96 \end{array}$$

- For *imposed axial displacement* and *partial contact*:

(6) $$\begin{array}{l} K_{{}_{\mathrm{IA},u\;\left[\mathrm{partial}\;\mathrm{contact}\right]}}^{*}=-33.68{\left(d/D\right)}^{3}+48.23{\left(d/D\right)}^{2}\left(\varepsilon /D\right)-82.67\left(d/D\right){\left(\varepsilon /D\right)}^{2}+\\ +97.13{\left(\varepsilon /D\right)}^{3}+58.06{\left(d/D\right)}^{2}-52.21\left(d/D\right)\left(\varepsilon /D\right)+14.00{\left(\varepsilon /D\right)}^{2}-\\ -37.80\left(d/D\right)+25.18\left(\varepsilon /D\right)+9.60\\ K_{{}_{\mathrm{IB},u\;\left[\mathrm{partial}\;\mathrm{contact}\right]}}^{*}=-34.28{\left(d/D\right)}^{3}-34.99{\left(d/D\right)}^{2}\left(\varepsilon /D\right)+164.47\left(d/D\right){\left(\varepsilon /D\right)}^{2}-\\ -37.45{\left(\varepsilon /D\right)}^{3}+60.03{\left(d/D\right)}^{2}+7.27\left(d/D\right)\left(\varepsilon /D\right)-28.95{\left(\varepsilon /D\right)}^{2}-\\ -33.50\left(d/D\right)-8.85\left(\varepsilon /D\right)+7.01 \end{array}$$

- For *remote tensile loading* and *no contact*:

(7) $$\begin{array}{l} K_{{}_{\mathrm{IA},F\;\left[\mathrm{no}\;\mathrm{contact}\right]}}^{*}=-27.99{\left(d/D\right)}^{3}+168.96{\left(d/D\right)}^{2}\left(\varepsilon /D\right)+67.03\left(d/D\right){\left(\varepsilon /D\right)}^{2}+\\ +104.39{\left(\varepsilon /D\right)}^{3}+54.08{\left(d/D\right)}^{2}-252.88\left(d/D\right)\left(\varepsilon /D\right)-45.84{\left(\varepsilon /D\right)}^{2}-\\ -36.00\left(d/D\right)+98.11\left(\varepsilon /D\right)+8.89\\ K_{{}_{\mathrm{IB},F\;\left[\mathrm{no}\;\mathrm{contact}\right]}}^{*}=-4.85{\left(d/D\right)}^{3}-174.58{\left(d/D\right)}^{2}\left(\varepsilon /D\right)-160.13\left(d/D\right){\left(\varepsilon /D\right)}^{2}+\\ +40.98{\left(\varepsilon /D\right)}^{3}+14.57{\left(d/D\right)}^{2}+262.86\left(d/D\right)\left(\varepsilon /D\right)+93.25{\left(\varepsilon /D\right)}^{2}-\\ -14.63\left(d/D\right)-101.65\left(\varepsilon /D\right)+5.28 \end{array}$$

- For *remote tensile loading* and *partial contact*:

(8) $$\begin{array}{l} K_{{}_{\mathrm{IA},F\;\left[\mathrm{partial}\;\mathrm{contact}\right]}}^{*}=-47.88{\left(d/D\right)}^{3}+112.10{\left(d/D\right)}^{2}\left(\varepsilon /D\right)-255.45\left(d/D\right){\left(\varepsilon /D\right)}^{2}+\\ +218.48{\left(\varepsilon /D\right)}^{3}+77.09{\left(d/D\right)}^{2}-92.64\left(d/D\right)\left(\varepsilon /D\right)+64.68{\left(\varepsilon /D\right)}^{2}-\\ -47.80\left(d/D\right)+33.90\left(\varepsilon /D\right)+11.56\\ K_{{}_{\mathrm{IB},F\;\left[\mathrm{partial}\;\mathrm{contact}\right]}}^{*}=-33.40{\left(d/D\right)}^{3}-47.91{\left(d/D\right)}^{2}\left(\varepsilon /D\right)+265.87\left(d/D\right){\left(\varepsilon /D\right)}^{2}-\\ -31.95{\left(\varepsilon /D\right)}^{3}+59.13{\left(d/D\right)}^{2}+2.32\left(d/D\right)\left(\varepsilon /D\right)-67.91{\left(\varepsilon /D\right)}^{2}-\\ -32.22\left(d/D\right)-4.40\left(\varepsilon /D\right)+6.43 \end{array}$$

### 3.2. Fatigue Crack Propagation Paths

**Figure 6:**
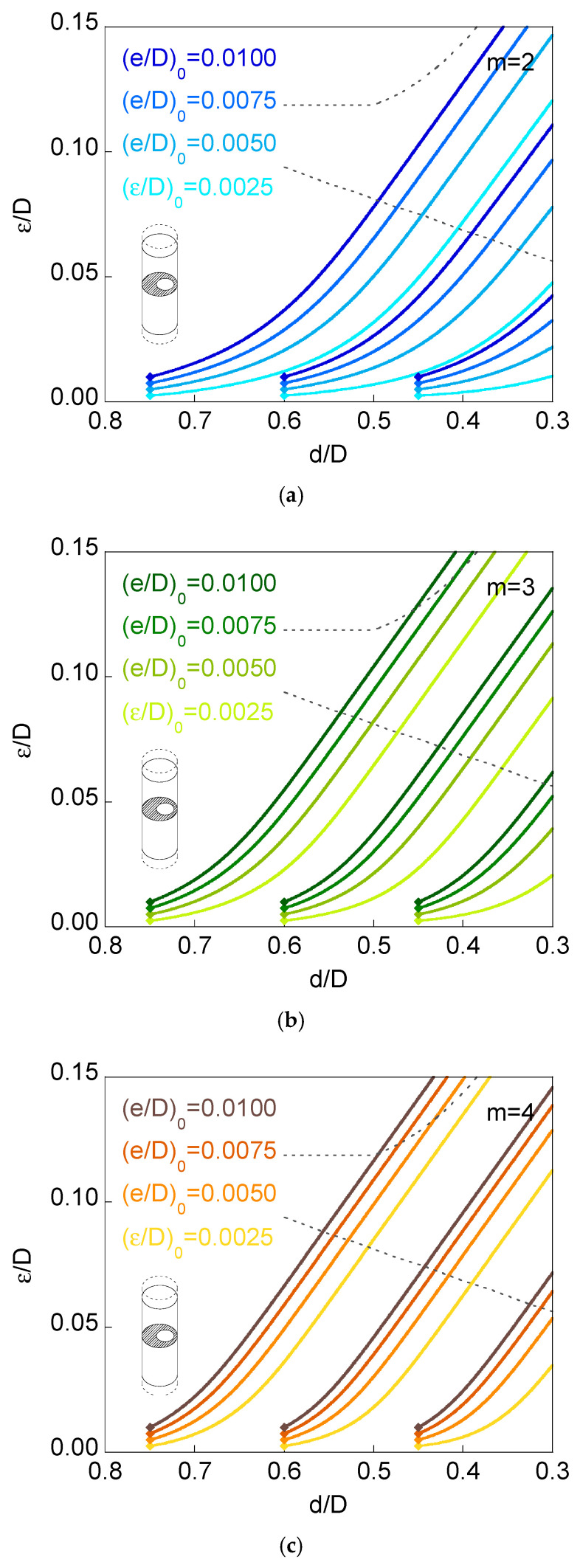
Curves *ε*/*D* vs. *d*/*D*, fatigue propagation for *imposed axial displacement*: (**a**) *m* = 2; (**b**) *m* = 3; (**c**) *m* = 4.

**Figure 7:**
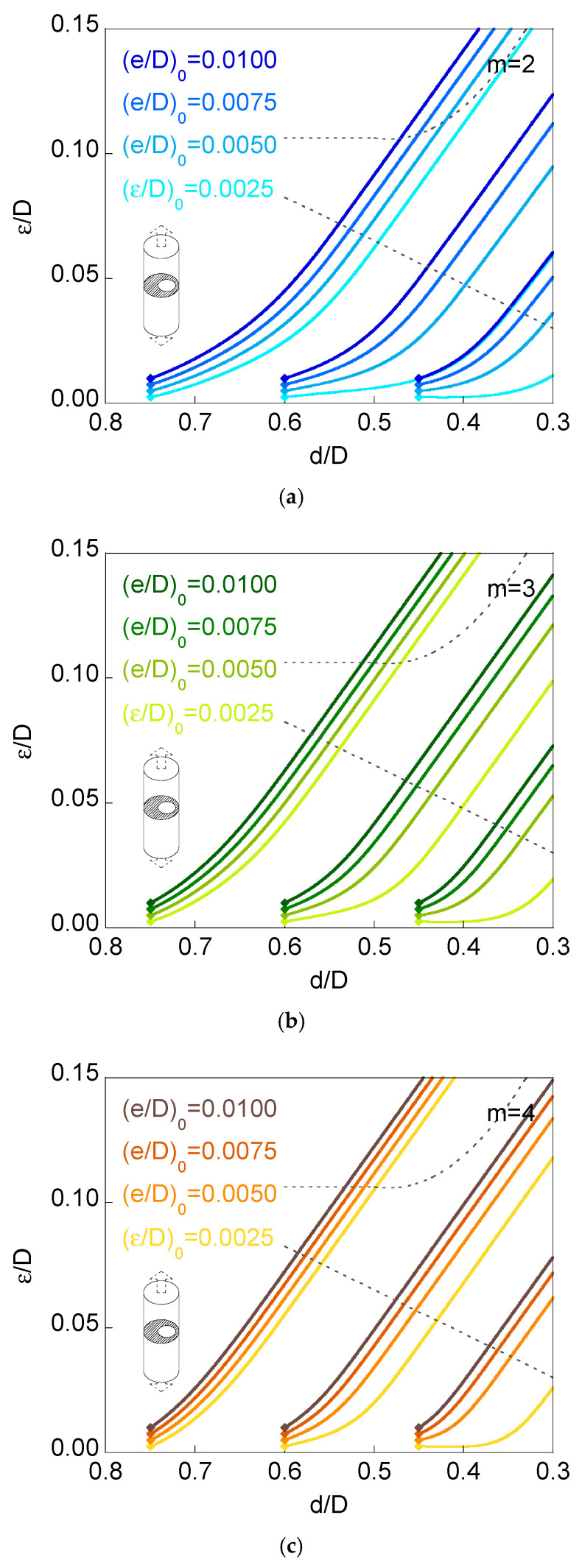
Curves *ε*/*D* vs. *d*/*D*, fatigue propagation for *remote tensile loading*: (**a**) *m* = 2; (**b**) *m* = 3; (**c**) *m* = 4.

Figure 6 and Figure 7 show the curves representing eccentricity *versus* ligament diameter (plots *ε*/*D* vs. *d*/*D*) for fatigue propagation of annular cracks, with three values for the initial relative diameter of the ligament (*d*/*D*)_0_ = {0.75, 0.60, 0.45} and as many as four levels of initial relative ligament eccentricity (*ε*/*D*)_0_ = {0.0025, 0.0050, 0.0075, 0.0100},n round bars (cylinders) subjected to either imposed axial displacement or to remote tensile loading, for three very different materials with Paris exponents *m* = {2, 3, 4}. These materials are representative of a wide range of metals and alloys that usually are the constituents of bars, chords, rods, shafts, strands, and wires, all of them having a round shape (i.e., cylinders) and working under pure tensile conditions.

The six plots in Figure 6 and Figure 7 show a general trend of decreasing relationship between the ligament eccentricity and the ligament diameter (it must be taken into account that the horizontal axes of the six plots in Figure 6 and Figure 7 are expressed in *decreasing units*). Therefore, as the ring-shaped outer crack grows towards the inner region of the cylinder, the ligament diameter decreases, whereas the ligament eccentricity (that is a measure of the full specimen eccentricity) increases, i.e., the specimen deviates from the axisymmetric case.

Another general trend of the six plots in Figure 6 and Figure 7 is the shape of the curves. All plots exhibit a curvilinear aspect at the beginning (low sample eccentricities and high ligament diameters), whereas after this initial period, all plots tend to a quasi-linear shape, i.e., the quasi-linear relationship between eccentricity and ligament diameter for the final stages of fatigue crack growth, i.e., for the highest levels of eccentricity itself.

The grey dashed lines in the six plots in Figure 6 and Figure 7 correspond to the beginning of the appearance of *partial contact* or *full contact* between the crack faces (Figure 5), a phenomenon occurring for lower values of relative eccentricity in the case of remote tensile loading than in the situation of imposed axial displacement, due to a bending effect in the sample in the first case, as discussed in previous sections of the present paper and in reference [13].

**Figure 8:**
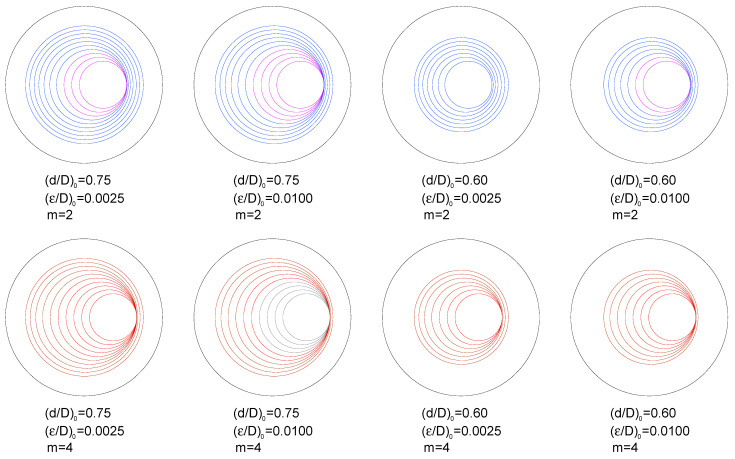
Crack fronts for *imposed axial displacement*; *m* = 2: *no contact* in blue, *partial contact* in pink, and *full contact* in grey; *m* = 4: *no contact* in brown, *partial contact* in red, and *full contact* in grey.

**Figure 9:**
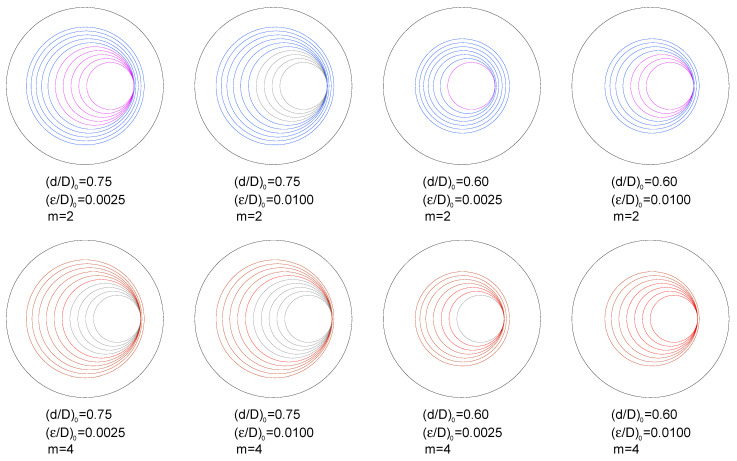
Crack fronts for *remote tensile loading*; *m* = 2: *no contact* in blue, *partial contact* in pink, and *full contact* in grey; *m* = 4: *no contact* in brown, *partial contact* in red, and *full contact* in grey.

Figure 8 and Figure 9 show the *fatigue crack paths* in the form of *circular crack front evolution* for given relative diameters of the ligament (from an initial value up to 0.30 with increments of 0.05) during fatigue crack growth from the initial geometries with two initial ligament diameters (*d*/*D*)_0_ = {0.75, 0.60} and two initial ligament eccentricities (*ε*/*D*)_0_ = {0.0025, 0.0100}. The crack fronts in blue (*m* = 2) and brown (*m* = 4) indicate that *there is no contact between the crack faces*, whereas the crack fronts in pink (*m* = 2) and red (*m* = 4) indicate that there is partial contact between the faces of the crack. Finally, the crack fronts drawn in dark grey colours indicate that full contact takes place between the crack faces.

The crack growth caused by fatigue obliges the relative eccentricity of the ligament to increase when the net diameter of the specimen (i.e., the ligament diameter) diminishes during the propagation phase. In addition, the values of the relative eccentricity during the crack advance are higher in the following three situations:

(i) When the external action over the cylindrical specimen (round bar containing a ring-shaped external circumferential crack) is a remote tensile loading instead of an imposed axial displacement (in the latter case, boundary conditions prevent sample bending).

(ii) With the increase in the Paris exponent *m* characteristic of the material behaviour, i.e., the higher the exponent *m* of the Paris Law (linked with a more accelerated fatigue crack growth), the higher the specimen eccentricity.

(iii) When the initial relative eccentricity of the ligament increases (for a given initial relative diameter), i.e., an elevated initial eccentricity promotes the further increase in the eccentricity of the sample even more.

The curves *ε*/*D*–*d*/*D* for different initial relative eccentricities of the ligament, with a given initial relative diameter, are closer to each other with the increase in the initial relative diameter of the ligament and when the initial relative eccentricities are more elevated. In addition, in the case of high initial relative diameters of the ligament, (*d*/*D*)_0_ = 0.75, the curves for different initial eccentricities become closer when the external action consists of remote tensile loading. On the other hand, in the opposite case of low initial relative diameters of the ligament, (*d*/*D*)_0_ = 0.45, such curves become closer when the external action consists of imposed axial displacement.

During fatigue crack propagation, all curves representing specimen eccentricity (or ligament eccentricity) as a function of ligament diameter, i.e., plots *ε*/*D versus d*/*D* tend towards quasi-straight lines that are, in addition, quasi-parallel between them. On assuming a strictly linear shape, all plots are strictly parallel, thereby sharing a common slope for a given material, i.e., a unique slope for a specific Paris exponent *m*, and different for distinct materials (distinct Paris *m* exponent).

The aforesaid equal slope of the *ε*/*D*–*d*/*D* plots corresponds to the growth when point B at the crack front remains immobile (the instant from which the supposition of a maintained circular crack front is impossible). Nevertheless, it is from the beginning of partial contact (or even before for the materials with the most elevated Paris *m* exponents) that the curves *ε*/*D*–*d*/*D* become straight lines, as observed in Figure 6 and Figure 7.

The present results are fully general for any CCRB specimen since a wide range of cracked geometries with very different initial depths of the external (outer) ring-shaped circumferential crack in the round bar were considered. With regard to the material, numerical results are only dependent on the Paris exponent *m* of the material. As a range *m* = {2,3,4} was considered, the results of the present paper are fully applicable to a really wide range of metallic materials.

The results are also applicable to environmentally assisted fatigue or corrosion-fatigue processes of metals and alloys by using a modified exponent of the Paris law for the situation of corrosion-fatigue, i.e., affected by the presence of a harsh or aggressive environment promoting an accelerated fatigue crack growth rate.

## 4. Conclusions

The following conclusions may be drawn regarding fatigue crack propagation paths in eccentric circumferentially cracked round bar (CCRB) specimens under tensile cyclic (fatigue) loading with remote tensile loading and imposed axial displacements:

(i) Closed-form stress intensity factor (SIF) solutions are provided for an eccentric outer (external) crack located in circumferentially cracked round bars (CCRB) under axial tensile loading in the form of remote tensile loading or imposed axial displacement.
(ii) Such SIF solutions are provided in the form of third-degree polynomial expressions as a function of the ligament diameter and the specimen eccentricity (both in dimensionless terms), covering a wide range of CCRB geometries.
(iii) The fatigue propagation paths associated with eccentric circumferential cracks located in round bars under tensile cyclic loading produce a clear augmentation of the eccentricity of the resistant ligament of the bar.
(iv) The described phenomenon is more intense when there is an increase in the initial relative eccentricity (for the same given initial relative diameter) and in the Paris exponent *m* characteristic of the material.
(v) In the eccentric CCRBs in which a remote tensile loading is applied, the increase in specimen eccentricity is higher than in those bars subjected to imposed axial displacement due to a bending effect.
(vi) The reason for the aforesaid higher increase in specimen eccentricity in the case of remote tensile loading is the external constraint (boundary condition) created by the imposed axial displacement, thereby preventing specimen bending.
(vii) The plots’ relative eccentricity versus relative diameter (*ε*/*D*–*d*/*D*) are straight lines of equal slope from the beginning of partial contact, or even before for the materials with more elevated exponent *m* of the Paris law.
(viii) Results possess a high level of generality: the provided closed-form SIF solutions (analytical expressions) are applicable to any fracture mechanics, damage tolerance, and structural integrity analyses in which CCRB geometries are involved.
